# Inescapable Stress Changes Walking Behavior in Flies - Learned Helplessness Revisited

**DOI:** 10.1371/journal.pone.0167066

**Published:** 2016-11-22

**Authors:** Sophie Batsching, Reinhard Wolf, Martin Heisenberg

**Affiliations:** Rudolf-Virchow-Center, University of Wuerzburg, Germany; Tohoku University, JAPAN

## Abstract

Like other animals flies develop a state of learned helplessness in response to unescapable aversive events. To show this, two flies, one 'master', one 'yoked', are each confined to a dark, small chamber and exposed to the same sequence of mild electric shocks. Both receive these shocks when the master fly stops walking for more than a second. Behavior in the two animals is differently affected by the shocks. Yoked flies are transiently impaired in place learning and take longer than master flies to exit from the chamber towards light. After the treatment they walk more slowly and take fewer and shorter walking bouts. The low activity is attributed to the fly's experience that its escape response, an innate behavior to terminate the electric shocks, does not help anymore. Earlier studies using heat pulses instead of electric shocks had shown similar effects. This parallel supports the interpretation that it is the uncontrollability that induces the state.

## Introduction

An animal may have established a behavioral response to cope with a recurrent dangerous or stressful event in the outside world. Changes of external conditions can be stressful, particularly, if they turn from controllable to uncontrollable. The significance of uncontrollability in behavior has been introduced as 'learned helplessness' by Seligman & Maier [1] who showed that dogs, after being extensively exposed to uncontrollable electric shocks, later failed to learn to escape the foot shocks in a shuttle box experiment. As J. H. Byrne [2] put it: "The term learned helplessness is used to refer to any behavioral or physiological consequence of exposure to an aversive event that is produced not by the event itself but by the organism’s lack of behavioral control over the event." In flies (*Drosophila melanogaster)* learned helplessness was first observed by Brown et al. [3] and later investigated in more detail by Yang et al. [4]. The latter authors used the so called heat box [5] in which they could expose single flies to a random sequence of heat pulses and measure their locomotor behavior. Flies out of control of the aversive events showed longer escape latencies, transiently impaired learning and low activity in walking.

In the present study we wanted to characterize learned helplessness in flies more closely. The early work on vertebrates had applied electric shocks as stressful situations. Their effects persisted for a long time and influenced other behaviors such as learning ability, sexual behavior, sleep, aggression and even immune status [6], [7]. We wondered whether the learned helplessness syndrome, introduced by Yang et al. [4], [8] via heat stress, could be reproduced with electric shocks. We wanted to know how the yoked flies coped with uncontrollable electric shocks and how, subsequently, other behaviors such as courtship, walking in an open field and learning ability were influenced by the experience of uncontrollability. We finally asked whether social isolation after hatching, another treatment known to affect the control of walking behavior in flies [9], would interfere with learned helplessness.

## Materials and Methods

### Flies

*Drosophila melanogaster* (wild type Canton S) were kept at 25°C on standard food on a 12 h light/dark cycle with 60% relative humidity. Flies were tested when they were 3–5 days old. Immediately before the test, flies were transferred to a fresh food vial to avoid grooming behavior during the experiments. For the courtship experiment male flies were collected one day after eclosion and kept in small groups until testing. For social isolation flies were transferred to small food vials, one fly per vial, at 1–2 hours after eclosion.

### Setup

#### Shock box

**Fig 1:**
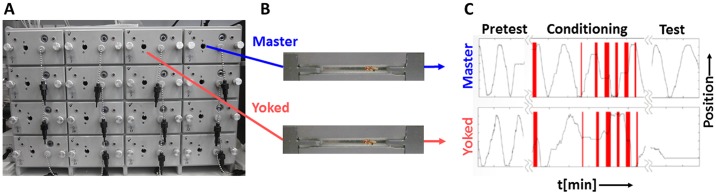
Shock box. (A) Front panels of 16 boxes. (B) Two chambers, each with a fly inside. (C) In each chamber the position of the fly is tracked. Time periods of electric shocks in red. For more details see Material & Methods.

The experimental setup consisted of 16 shock boxes (Fig 1) built in the workshop of the Biocenter, Würzburg. Each box contained a chamber (29 mm x 4 mm x 2 mm) to house a single fly. Floor and ceiling of the chamber were covered by gold-plated electric grids to deliver electric shocks (80 V peak to peak, 4 Hz, AC) to the fly. Side walls were of glass. Chambers were illuminated from one side by IR light. A linear electro-optical position detector was mounted oppositely to track the fly’s shadow. Position was resolved with 0.2 mm resolution and recorded at 10 Hz. Boxes were connected to a computer and operated by a custom made software written by Andreas Eckart (Biocenter, Wuerzburg). Temporal sequence of fly position and electro-shock information were stored and later processed with a self-written evaluation software. Temperature in the chamber was kept at 24°C. Chambers were humidified by a slow air current running through a wash bottle before and after every experiment.

#### No-idleness experiment

The master fly received electric shock in case it rested for more than 1 s. The punishment was immediately switched off if the fly resumed walking. Whenever the master fly got shocked the yoked fly got shocked as well, independent of its locomotor activity. The experiment consisted of 1 min pretest, 20 min conditioning, followed by 10 min test, unless otherwise specified. Only flies showing locomotion during the first minute of the experiment were included in the data evaluation.

#### Handling

For transfer to the shock box, flies were sucked up into an aspirator and blown into the chamber. For the free-walk experiments (below), flies had to leave the shock box after the 20 min conditioning and walk into a plastic vial, from where they were transferred to the arena. For the courtship experiments (below) the same procedure was used to transfer a male fly to the courtship chamber. For transfer to the heat box flies were transported in a vial covered by black foil to keep them in constant darkness.

#### Place learning

Whenever the fly entered one side of the chamber of the shock box it received an electric shock (100 V peak to peak, 4 Hz, AC). The 'punished' side was alternated from fly to fly to avoid any side biases in the averages. Only flies which crossed the midline during the 1 min pretest and received at least one shock during the first training were included in the evaluation. The performance index was calculated subtracting the time the fly spent on the punished side from the time spent on the unpunished side divided by the total time. The experiment consisted of 1 min pretest, 1 min training 1, 1 min test 1, 1 min training 2, 1 min test 2, 1 min training 3 and 2 min post-test.

#### Free-walk arena

To record walking trajectories in an open field we built a free-walk arena. It consisted of a modified Petri dish (Ø = 86 mm, h = 4 mm) covered by a glass lid and placed on white paper. The arena was surrounded by an opaque acrylic glass cylinder (Ø = 125 mm, h = 130 mm) with four vertical black stripes (h = 130 mm, w = 15 mm) spaced at 90° on its inner surface. The cylinder was again surrounded by another translucent milk-glass cylinder (Ø = 295 mm, h = 240 mm). The inner surface of the outer cylinder carried three horizontally running rows of LEDs (Flex Strip, Synergy 21, Germering, Germany) to illuminate the arena. Trajectories were tracked for 10 min at 40 Hz using a standard USB webcam (Logitech, C500), stored on a computer and later evaluated using a custom made program, written by S. Koenig (Rudolf Virchow Center, Wuerzburg). Walking activity in the free-walk arena, was defined as a displacement velocity of d >/ = 4 mm/s. With the definition of walking activity in the shock box (d >/ = 0.6 mm/s) absolute values were larger but the conclusions were the same.

#### Mating chambers

Courtship was recorded in daylight at 24°C room temperature using a "courtship wheel" with four chambers (Ø = 8 mm, h = 6 mm; v = 0.3 cm^3^), modified after Hotta and Benzer [10] built in the workshop of the Biocenter, Würzburg and a USB webcam (Logitech, C500). Videos were later analyzed by inspection. Courtship and wing vibration indices were calculated as % of time flies showed the respective behavior.

### Statistical Analysis

Data were tested for normal distribution using a Kolmogorov-Smirnov test. Bonferroni corrections were used for multiple comparisons. If no normal distribution could be assumed, a Mann-Whitney test was used to test two groups against each other. Comparison of more than two values was achieved by a one-way ANOVA with Holm-Sidak’s multiple comparisons test, if the data were normally distributed and otherwise with a Kruskal-Wallis test with Dunn’s test for multiple comparisons (* = p < 0.05, ** = p < 0.01, or *** = p < 0.001).

## Results

Flies were tested in a small box (shock box; Fig 1) [11]. It closely resembled the heat box [4] except that the flies could be exposed to electric shocks instead of heat pulses. As originally introduced by Seligman and Maier [1] we used the triadic design and divided the experimental animals into three groups: 'master', 'yoked' and controls. In the standard experiment the master flies received electric shocks during rest periods. Whenever the master fly got shocked, the corresponding yoked fly also got an electric shock of the same duration, independent of what it was doing at this moment. When the master fly resumed walking, the shock went off for both, master and yoked. Control flies did not get any shocks.

### Uncontrollable Electric Shocks Have a Lasting Effect on Walking Behavior

Conditioning with heat pulses [4] had shown that yoked flies became less active, reduced walking speed and paused less often but longer, compared to master flies. We have now observed similar changes in walking behavior exposing flies to electric shocks.

**Fig 2:**
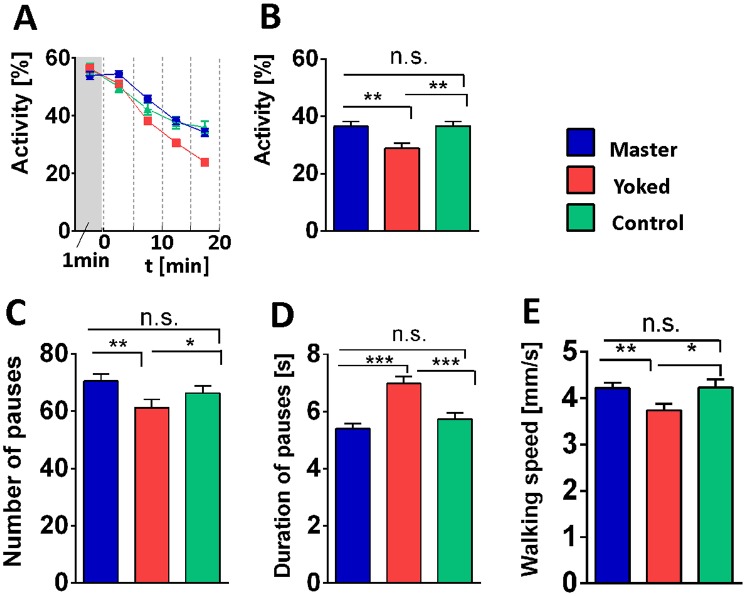
Modulation of walking behavior in the shock box. **(A)** Pretest and conditioning. No shock pulses during pretest (1 min). Conditioning data are pooled in 5 min bins. Master (blue); yoked (red); controls (turquoise). From the fifth minute on, yoked flies become less active than master and control flies. **(B-E)** 10 min test without shock. **(B)** Activity. Yoked flies remain less active than master or control flies. No significant difference between the latter two. **(C)** Yoked flies make fewer pauses than master or control. **(D)** Pauses are longer in yoked flies than in the other two groups. **(E)** Control and master flies walk faster than yoked flies. Master/yoked: n = 220, control: n = 174. ***p < 0.001; **p < 0.01; n.s., p > 0.05.

We measured the fly's position in the shock box in time and made the master flies switch the current on 1 s after they stopped and turn it off immediately when they resumed locomotion. The yoked flies without any contact to their respective master, received the same shocks at the same time without having any control. As a third group we included flies that did not receive any shocks while in the box (box controls). Fig 2A shows walking activity (% time spent walking) of the 3 groups during the 20 min conditioning phase.

The conditioning phase was followed by a 10 min test without electric shocks (Fig 2B–2E). The same four parameters of walking behavior were evaluated as in the heat box: walking activity (% time spent walking), the number and duration of pauses and walking speed (distance covered during walking). In all four parameters the electric shocks had an after-effect in the yoked flies. These were less active, walked more slowly and made fewer but longer pauses than control flies without electric shock. Master flies did not differ from controls. These results closely resemble those in the heat box mentioned above [4].

For a close comparison of the results in the two boxes, we conducted an experiment in the shock box with exactly the same time course as the one in the heat box (10 min conditioning followed by 30 s test; data not shown). The main difference between heat and electric shocks was that in the shock box flies were less active due to longer but about the same number of pauses. Despite this disparity, the master/yoked difference was as described: With both stressors yoked flies walked more slowly and showed longer and fewer rest periods than master flies.

Finally, with heat pulses and electric shocks the effects of uncontrollability were more pronounced in females than in males (female/male comparison for heat in [4], for electric shocks in S1 Fig).

Given that the perception of heat and electric shock is mediated by different peripheral receptors and neural pathways [12], these similarities suggest that it was not the heat or electric shocks *per se* but their uncontrollability in the yoked flies that modulated the walking behavior.

### *Drosophila* in the Shock Box Has an Innate Escape Response to Electric Shock

**Fig 3:**
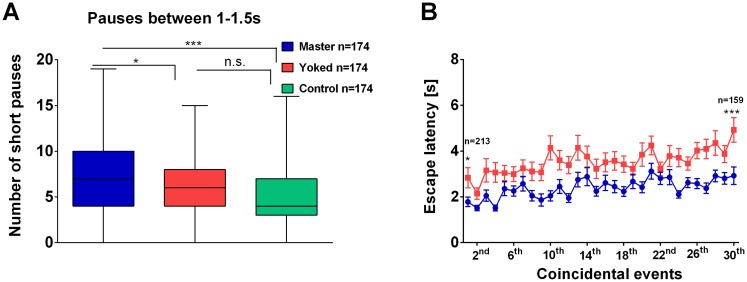
Response to electric shock. **(A)** Number of short pauses (duration 1–1.5 s) in the first 2 min of conditioning. All master flies (blue) are hit by a shock at 1 s of the pause. They take more short pauses than yoked flies (red) or controls (turquoise). Yoked flies have a low probability to be hit by the shock at an arbitrary time during the pause. Their short pauses are not significantly more frequent than in the controls. Non-parametrical presentation of the data. Data in the box represent the 25% and 75% quartiles, while the error bars represent the distribution down to the minimum and up to the maximum values of the dataset. Number of flies in each group: n = 174. **(B)** Escape latency. Time the fly needs to start walking after the onset of an electric shock. Coincidental events are extracted from the position/time traces (and averaged over flies) in which not only the master fly but also the yoked fly sits during the onset of the shock and the yoked fly is hit only once during that rest period. Yoked flies hit by electric shock during rest period take longer to resume walking than master flies. Female master/yoked pairs for first coincidental event, n = 213 and for the 30^th^ event n = 159. ***p < 0.001; **p < 0.01; n.s., p > 0.05.

The rationale of the master/yoked comparison in the above experiments was to make the treatment of the two groups of flies as similar as possible. However, the temporal relation between electric shocks and rest periods remained different between master and yoked flies. Yoked flies could be hit by shocks during rest and during walking. It could not be excluded that shocks (or heat pulses) during walking but not during rest caused the changes in walking behavior observed. We therefore looked more closely at the conditioning process to see whether it was indeed the uncontrollability that mattered. Evidently, electric shock is an aversive stimulus as shown, for instance, in olfactory learning [13] and place learning in the shock box [11], where flies show avoidance of the shock-associated side. However, does a fly at rest in the shock box start walking in response to a shock? Does it try to escape? As in the present experiments electric shock for the master flies started 1 s after locomotion stopped, we asked whether short pauses lasting between 1 s and 1.5 s were more frequent in master than in yoked or control flies. Indeed, short pauses were the most frequent in master, less frequent in yoked and control flies (Fig 3A; data shown for the first 2 min; similar result for whole 20 min period, not shown). The reduced frequency in yoked flies indicates that in master flies some of the short pauses must have been escape responses.

### Different Latencies of the Escape Response in Master and Yoked Flies at Rest

The master fly hit by an electric shock at rest terminates it via its escape response. Occasionally also the yoked fly is hit by an electric shock at rest. Does it also try to terminate it via an escape response? To answer this question we measured in master and yoked flies the latency between the onset of the shock at rest and the initiation of walking (Fig 3B). We searched for events in which the electric shock initiated by the master caught the yoked fly during a pause and we measured for these coincidental events how long it took the master and yoked flies to resume walking. Each master/yoked pair generated a series of such events and we averaged these escape latency values over all the pairs according to the rank of the respective event in the series. Coincidental events were included only if the yoked flies had terminated their rest period before a second electric shock arrived. Latencies were markedly longer in yoked than in master flies (Fig 3B). Given the fewer short pauses this indicates that yoked flies reduced the frequency of escape responses, presumably, because these were ineffective. This would imply that a fly monitors the effectiveness of the response and modulates its outcome expectation [14] accordingly.

### Learned Helplessness Is Reversible

**Fig 4:**
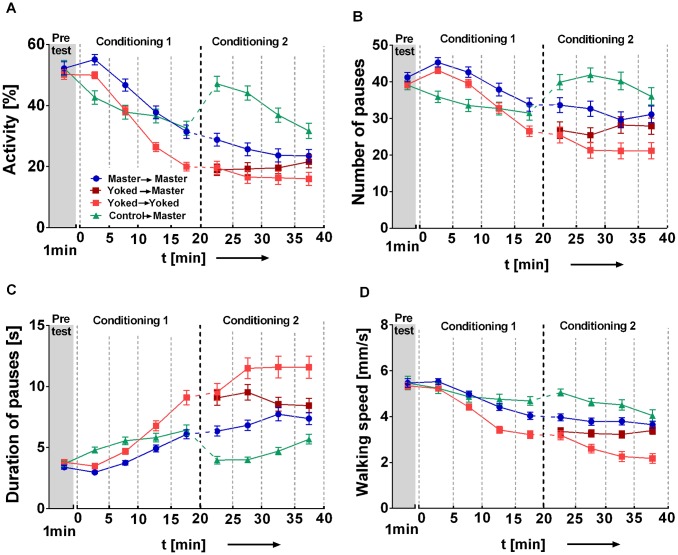
Motivational states change with changing experience. Control-to-master (turquoise), master-to-master (blue), yoked-to-master (light red in conditioning phase 1, dark red in conditioning phase 2), and yoked-to-yoked (light red) flies. Pretest (1 min) and conditioning (pooled in 5 min bins). Walking activity is defined as in Fig 2. Conditioning 1: Master/yoked pairs receive shock pulses if master fly stops walking. Yoked-to-master and yoked-to-yoked flies are pooled. Control flies receive no shock pulses at all. Conditioning 2: Control over the shocks is given to master-to-master, yoked-to-master and control-to-master flies. Yoked-to-yoked flies stayed yoked. We split yoked flies from conditioning 1 into yoked-to-master and yoked-to-yoked flies. **(A)** Walking activity during 1 min pretest and 40 min conditioning phase: During the first five minutes all three groups differ in activity. Master-to-master and control-to-master flies remain more active in the end of the first 20 min conditioning. Starting from min 20, yoked-to-master flies need several min to align to master-to-master flies. Control-to-master flies show an increasing activity level with the onset of electric shock (min 15–20). **(B)** Number of pauses. Master-to-master and control-to-master flies make more pauses during min 15–20 when compared to yoked. In min 30–40, yoked-to-master flies align to master-to-master flies. **(C)** Duration of pauses. During min 5–20, yoked flies make longer pauses than the compared groups. During min 20–30, yoked-to-master make longer pauses than master-to-master flies. In the subsequent 10 min, yoked-to-master flies differ from control-to-master and yoked-to-yoked, but not from master-to-master flies. **(D)** Walking speed. Starting from the 5th min, yoked flies walk more slowly than the compared groups. In min 35–40, yoked-to-master flies align their walking speed to master-to-master flies. Master-to-master, n = 102; yoked-to-master, n = 102; control-to-master, n = 88 and yoked-to-yoked (n = 103) flies.

As shown above (Fig 2), during conditioning the walking activity of yoked flies dropped in comparison to the master and control groups and stayed low in the subsequent 10 min without electric shocks. In the following experiment we wanted to know how flexible the flies still are after the 20 min conditioning period. We therefore added a further 20 min period in which the flies of all three groups were conditioned as master flies (Fig 4A–4D). In a forth group the flies that had started as yoked, stayed yoked in the second part (light red). After the switch from control to master condition flies increased walking activity by 47% and reduced the mean duration of pauses from 6.4 to 3.9 s in the first 5 min of being masters. Remarkably, during that period the yoked-to-master flies were not distinguishable from the yoked-to-yoked flies. However, gradually they changed and approached the master-to-master flies in all four parameters (activity, speed, duration and number of pauses). Characteristically, the flies that stayed in the yoked mode for the 2^nd^ period walked more slowly, kept longer pauses and rested less frequently than the yoked-to-master flies. The latter obviously had noticed the change of the rules and resumed control.

### Uncontrollability State Suppresses Learning Behavior

Dogs are unable to escape from the electric shocks in a shuttle box, after having been extensively exposed to uncontrollable electric shocks ('learned helplessness'; [1]). Originally the authors assumed that the dogs had learned to be inactive and were thus not showing an escape behavior. Meanwhile they found that the failure in the shuttle box was due to passivity, not to associative learning [15].

**Fig 5:**
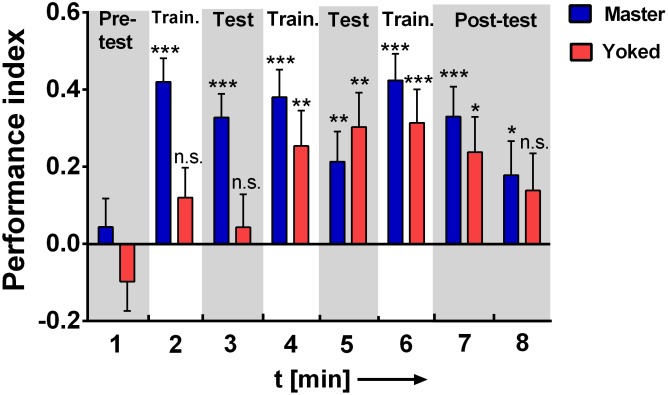
Impact of inescapable shocks on place learning. Place learning experiment subsequent to 20 min conditioning phase in the chambers. 1 min. measuring periods are shown. Positive performance indices indicate avoidance of the punished side (for more details see Material & Methods): In the first training and test the yoked (red) flies did not avoid the punished side while the master (blue) flies performed normally. During post-test avoidance of the punished side was less stable in yoked than in master flies. Master/yoked pairs, n = 58. ***p < 0.001; **p < 0.01; n.s., p > 0.05.

To characterize the effects of uncontrollability on learning behavior we tested master and yoked flies in place learning in the shock box [11]. Considering that after the 20 min conditioning phase some flies kept very long rest periods (Fig 2), we only included flies in the place learning experiment, if they (a) showed any walking during the 1 min pretest and (b) received at least one electric shock during the first training. Ten pairs had to be discarded due to these criteria. Pilot experiments had shown that in place learning flies expressed significant memory scores when conditioned with 100 V, 4 Hz (data not shown). Due to the criteria mentioned above, 58 master and yoked flies remained in the experiment and differed considerably in the first few minutes. While master flies performed about normally, yoked flies showed neither any avoidance of the shock-associated side of the chamber during the 1 min training, nor during the 1 min test after the shock had been turned off. The training/test procedure was repeated a 2^nd^ and 3^rd^ time. The yoked flies recovered and already in the 2^nd^ round showed significant avoidance and place learning. The memory, however, seemed to be less stable (Fig 5) as the score in the 2^nd^ min was not significant.

### Handling Does Not Abolish the State of Uncontrollability

**Fig 6:**
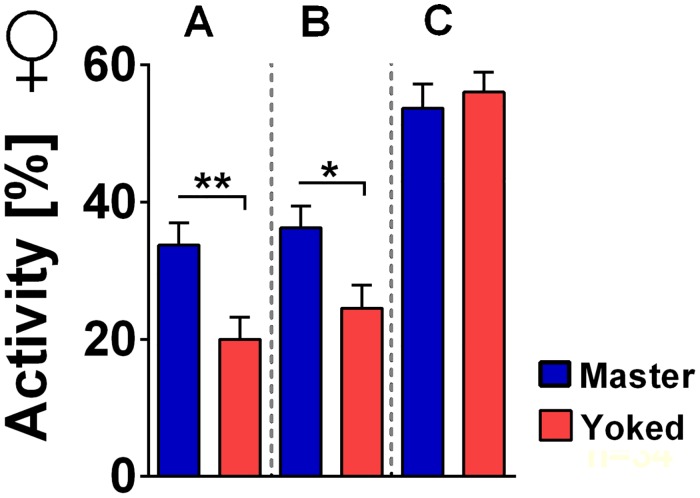
Effect of handling. Master (blue), yoked (red). Walking activity during 10 min test phase (no shocks) subsequent to 20 min of conditioning in the shock box. **(A)** No handling (master/yoked pairs: n = 38). **(B)** After handling (master/yoked pairs: n = 34). **(C)** Transfer to the heat box abolishes the uncontrollability effect (master/yoked pairs: n = 29). ***p < 0.001; **p < 0.01; n.s., p > 0.05.

For learned helplessness in mammals it had been reported that the respective motivational states persisted for a long time and interfered with other behavioral activities such as sexual behavior and food intake [16]. We wondered whether uncontrollability in the shock box would be context specific or would also show in other behavioral activities. For such experiments the flies would have to be transferred from the shock box to some other environment. We therefore tested whether already the handling had an impact on the motivational states. Flies after the 20 min conditioning phase were allowed to walk from the shock box into a dark transfer box, from where they were taken up into an aspirator and blown back into a shock box for the test phase. This was conducted under dimmed light conditions to avoid that the exposure to bright light could influence the behavior. Differences between master and yoked flies remained stable (compare Fig 6A and 6B). We also transferred some of the flies to a heat box which had similar size but no humidity control and no electric grids. Surprisingly, these changes already abolished the master/yoked differences (Fig 6C).

### Yoked Flies Need Longer than Master Flies to Exit the Box towards Light

**Fig 7:**
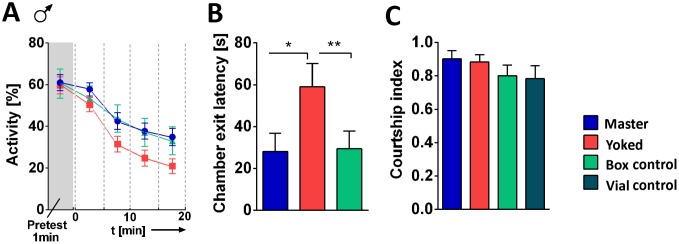
Male courtship and leaving the shock box. Male flies were conditioned for 20 min in the shock box and subsequently transferred to a courtship chamber where their courtship behavior was recorded for 10 min. In addition, the chamber exit latency was measured. **(A**) Male pretest and conditioning. Experimental conditions as in Fig 2: Starting from min 15 yoked (red) flies indicate a lower activity level, when compared to master (blue) or control (turquoise) flies. Master/yoked pairs, n = 20; box control, n = 22 **(B)** Chamber exit latency: Time needed to crawl out of the chamber into a brightly lit transport vial. Yoked flies took longer time than master and box control flies. Master, n = 38; yoked, n = 44; control, n = 41. **(C)** Courtship index (for more details see Material & Methods) during 10 min recording time: No differences were observed between master, yoked flies and box controls and vial controls (petrol blue). (Pairs: n = 20; box control: n = 22; vial control: n = 27). ***p < 0.001; **p < 0.01; n.s., p > 0.05.

From mice and flies it is known that exposure to stress leads to anxiety-like symptoms, as shown, for instance, in the so-called shelter paradigm, where the animals avoid exposure to light [17], [18]. In the present experiments we measured the time flies needed to leave the shock box after the conditioning. In this case the transfer box was translucent to potentially elicit positive phototaxis. Striking differences between the three groups, master, yoked and controls were found. Yoked flies took twice as long as master or control flies to walk out into the light (Fig 7B).

Note that in the experiments shown in Fig 7 we used males instead of females since we were planning to test master and yoked males in courtship. The characteristic differences between master and yoked flies in walking behavior as shown in Fig 2 were all found in males as readily as in females (Fig 7A for activity; more data in S1 Fig).

### Uncontrollability State Does Not Interfere with Courtship in Males

It has been shown in male rats, that exposure to a stressful situation decreases their sexual behavior e.g. [19] and in humans sexual dysfunction is a well-known symptom in depression [20]. We transferred a single master or yoked male to a small chamber where we recorded its courtship behavior in the presence of a virgin female and measured the courtship index, courtship latency, wing vibration index and wing vibration latency. In addition to the control flies that spent 20 min in the box without receiving shocks (box control), we compared males taken directly from food vials that had not experienced the 20 min period in the shock box (vial control) to exclude the highly restrained situation in chambers to be the cause for changes in subsequent behavior. In none of the parameters master, yoked and both control groups showed any differences. In Fig 7C just the courtship index is displayed (but see also S2 Fig). Their courtship index was the same.

### Electric Shock but Not Uncontrollability Has an Impact on Subsequent Open-Field Walking

**Fig 8:**
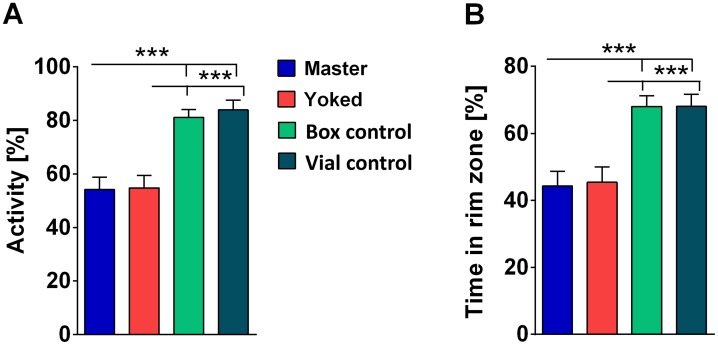
Open-field walking. Data are pooled for 10 min periods. **(A)** Walking activity during 10 min after transfer from 20 min conditioning in the shock box. Master (blue) and yoked (red) flies walk less than controls (box control: turquoise; vial control: petrol blue). **(B)** Time flies spend in the rime zone. Rim zone width is defined as the most peripheral 20% of the arena radius. Both control groups spend more time in the rime zone than master or yoked flies. No master/yoked difference. Master/yoked pairs, n = 20; box control, n = 20; vial control, n = 20. ***p < 0.001; **p < 0.01; n.s., p > 0.05.

In the shock box the flies are highly constrained. A more common procedure of testing walking behavior in animals is the open-field test. Hall [21] introduced it to study anxiety-like behavior in vertebrates. We transferred female flies after the 20 min conditioning to an open-field arena (diameter: d = 86 mm) and recorded their trajectories for 10 min. Master and yoked flies showed lower walking activity and spent less time in the rim zone compared to control flies that had been in the chamber without shock (box control) or to vial control flies (see above Fig 7). Interestingly, no difference was found between master and yoked flies (Fig 8A and 8B). Uncontrollability seemed not to matter for walking behavior in the new environment (open-field, light).

### Single-Reared Flies Walk More in the Shock Box and Are Less Affected by Uncontrollability

**Fig 9:**
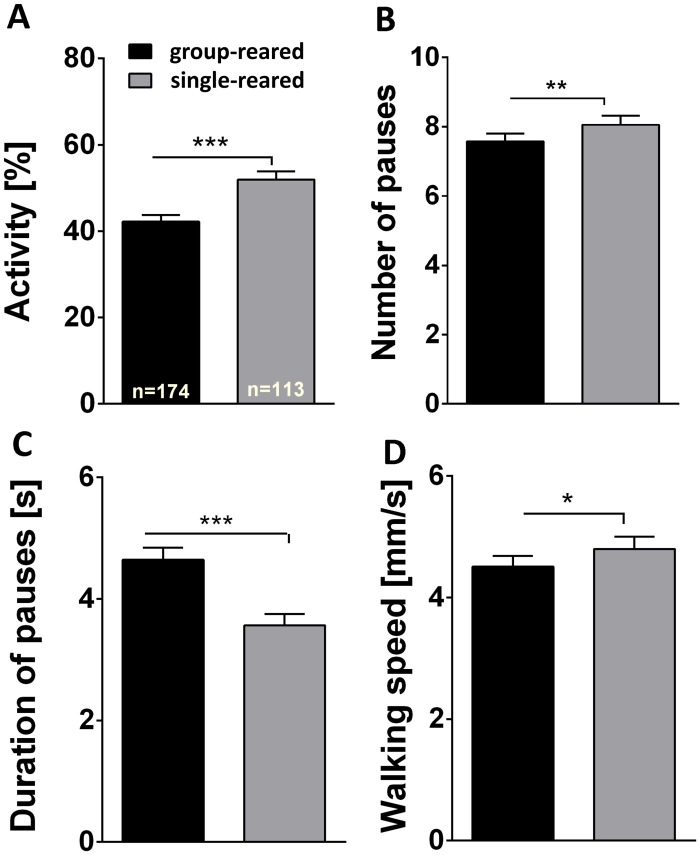
Impact of rearing conditions on walking behavior in the shock box. Walking behavior of group-reared (black) and single-reared (grey) female flies during 20 min in the shock box without electric shocks. Except for test duration, parameters evaluated are the same as in Fig 2B–2E. **(A)** Walking activity. Single-reared flies are more active than group-reared flies. **(B)** Number of pauses during 20 min. Single-reared flies make more pauses than group-reared flies. **(C)** Duration of pauses per minute. Single-reared flies make significantly shorter pauses when compared to group-reared flies. **(D)** Walking speed. Single-reared flies walk faster than group-reared flies. Group-reared, n = 174; single-reared, n = 113. ***p < 0.001; **p < 0.01; n.s., p > 0.05.

**Fig 10:**
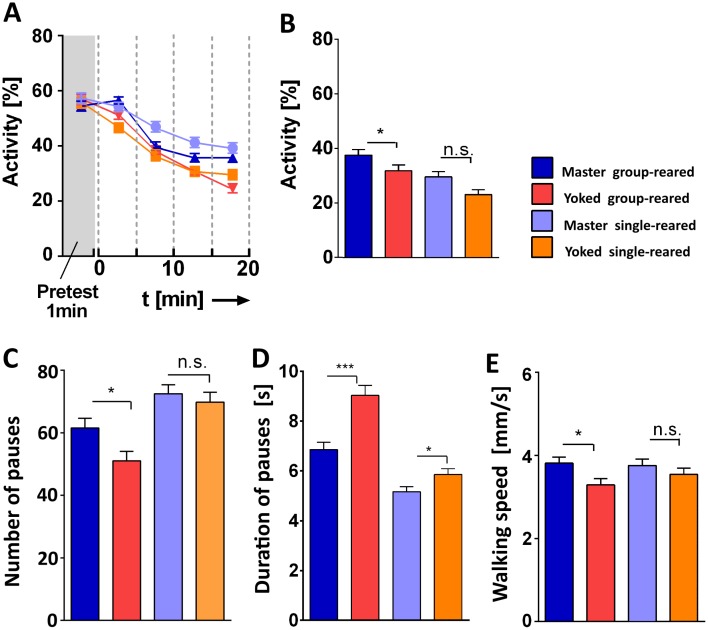
Impact of rearing condition on learned helplessness. Group-reared master (blue), group-reared yoked (red), single-reared master (light blue) and single-reared yoked (orange). No shock pulses during pretest (A) and test (B-E). Same parameters as in Fig 2. **(A)** Pretest and conditioning. Yoked flies become less active than their respective master flies. **(B)** During the 10 min test, group-reared yoked flies remain less active than group-reared master. No significant yoked/master difference between the single-reared flies. **(C)** Number of pauses. Group-reared yoked flies make fewer pauses than their respective master flies. No significant difference between single-reared flies. **(D)** Durations of pauses during test differ between the group-reared master and yoked flies. A small difference is also observed between single-reared master and yoked flies. **(E)** Walking speed during test: Group-reared master flies differ significantly from group-reared yoked flies while this difference is not significant in single-reared flies. Group-reared master/yoked, n = 118; single-reared master/yoked pairs, n = 103. ***p < 0.001; **p < 0.01; n.s., p > 0.05.

Keeping a fly in isolation for several days after hatching from the pupal case has pronounced effects on various behaviors, e.g. [22]. We wondered whether and how walking in the shock box was affected by this long-term experience. In the basic experiment of Fig 2 the flies were transferred from a group rearing situation to isolation in the shock box. Now these flies were compared to flies that had lived in isolation already for all their adult life. Surprisingly, flies reared in isolation were more active, walked faster and had more but shorter pauses (Fig 9A–9D). Once the conditioning started, these marked differences in walking did not show directly anymore (Fig 10A) but in the single-reared flies the master/yoked differences were less pronounced and did not last through the subsequent 10 min test period (Fig 10B–10E). Apparently, the single-reared flies were less affected by uncontrollability.

## Discussion

### Is Uncontrollability the Stressor?

One of the goals of this study was to confirm that learned helplessness in flies is indeed, what it had been called according to the earlier work in other animals (see Introduction). For this we needed to show that in the yoked flies the behavioral changes caused by the shocks were not caused by the shocks directly but by their uncontrollability. Recently Maier and Seligman [15] coined the term 'subjectively helpless' implying that the animal measures the outcome of its action, derives an outcome expectation for the future and compares it to the respective outcome expectation derived from similar events in the past. In this way it learns that its action is ineffective, that it is helpless.

In the present study both, master and yoked flies make use of what is offered by the experimental design, control and, respectively, uncontrollability. This is shown by the frequency of short pauses and the latency of escape responses (Fig 3A and 3B). Both results require outcome learning [14]. The flies measure the outcome and modulate the response frequency, if the outcome deviates from the outcome expectation. In other words, the yoked fly may be subjectively helpless. Whether all the other behavioral effects such as slower walking, fewer and shorter walking bouts, impaired learning and slower escape to light are all due to subjective uncontrollability, however, cannot be finally proven. Although considered very unlikely, it cannot be excluded that the shocks during walking cause some or all of the behavioral effects directly.

### Electric Shocks and Heat Pulses Cause the Same Symptoms

It was this limitation of the experimental design that had prompted us to repeat the experiment of Yang et al. [4] with electric shocks instead of heat pulses. The striking parallels in nearly all behavioral effects strongly support the assumption that it is the uncontrollability and not the stressor directly that modulates the behavior.

### Different Effects of Electric Shocks and Heat Pulses

Outside the realm of subjective helplessness heat and electric shocks do differ in some of their effects on fly locomotion in our experiments. For instance, at the onset of the conditioning phase with electric shocks there is a special arousal effect that is not observed with heat pulses. The effect is most apparent in control flies that after 20 min are switched to being masters (control-to-master; Fig 4A).

The finding that escape responses in the heat box are generally shorter than with electric shocks may indicate that the fly notices the gradual increase of temperature and tries to keep it from fully rising to its maximum.

Another difference is the activity right after transfer to the box, before the onset of conditioning. The lower activity in the shock box can be attributed to the high humidity. If this is switched off, walking activity rises to its level in the heat box (data not shown).

In the open-field test after conditioning in the shock box master and yoked flies show the same symptoms. Hence, in this case the effects can be attributed to the electric shocks rather than the uncontrollability. Heat was not tested. The confinement to the box as the cause can be excluded, since flies directly from the growth vial (vial control) behave like the controls from the shock box.

### Characterizing Learned Helplessness

Our study and that of Yang et al. [4] differ from most studies of learned helplessness on other animals in that the flies are exposed to aversive events only for short periods (10 or 20 min as opposed to several hours or days [23]). As might be expected the behavioral states induced are less stable. If after 20 min of uncontrollability control is re-installed (yoked-to-master), it takes the fly several minutes to adjust to the new condition. Under test conditions (no electric shocks) after 20 min of uncontrollability the state of less and slow walking persists for more than 10 min.

Interestingly, flies reared in social isolation seem to be stressed less by uncontrollability than group-reared flies. They show only weak 10 min after-effects (Fig 10). Possibly the transfer to isolation in the shock box is less stressful than for group-reared flies. In the first minute after transfer, their locomotion seems normal. They are as active as group-reared flies. Over 20 min without electric shocks, however, activity and walking speed decline substantially less in single-reared than in group-reared flies.

In the early experiments on learned helplessness [24] the dogs were conditioned and tested in very different surroundings [16]. From mice and rats learned helplessness is known to have many behavioral and physiological consequences, e.g. for food intake, social behavior and sleep [25]. In the present study the uncontrollability state appears to be strongly context dependent. It disappears in a different environment such as a free-walk arena, a courtship chamber or even a heat box. Whether this property distinguishes flies from mammals or whether it is due to the different experimental conditions, remains to be found out.

Handling alone seems not to affect the uncontrollability state. After the return to the shock box the same symptoms as without handling are observed (Fig 6). Conditioning in the heat box seems to be less resistant to handling (data in S3 Fig). In all these cases it cannot be excluded that with a longer or more severe conditioning regime the state of uncontrollability might still show. Recently, Ries [23] exposed flies to uncontrollable shaking (300 Hz) for 8 hours a day for 3 days and observed long lasting behavioral changes in different environmental situations such as delayed courtship.

## Conclusions

Flies exposed to aversive heat pulses or electric shocks in a small, dark chamber show 'learned helplessness', if their innate escape response that should protect them against such events, has no beneficial effect. The fly quickly finds out that escape responses are ineffective and reduces their frequency. In the time after the shocks while still in the box, the fly also reduces the frequency and duration of walking bouts as well as walking speed. It is impaired in a learning task with the aversive treatment as reinforcer and delays its exit from the box towards the light. No learned helplessness is observed after transfer to a new environment.

## Supporting Information

S1 Data Raw data to figs 1–10. (PDF)

S1 Fig Learned helplessness is more pronounced in females than in males. Females (black), males (light grey). Properties of walking behavior during 20 min conditioning and subsequent 10 min test. Data bars represent the respective difference (Δ) between master and yoked flies. For absolute values compare Fig 2A (females) and Fig 7A (males). The four groups of flies were measured side-by-side. Test parameters are defined as in Fig 2. For all of them differences of males are smaller than of females but only in one case significantly. **(A)** Activity during pretest and conditioning. Activity is shown in 5 min bins. **(B)** Activity during test: master male and female flies show higher activity level during test than yoked flies. **(C)** Number of pauses: Difference between master and yoked is more pronounced in females but does not differ significantly from males, while males do not show a statistically significant difference between master and yoked. **(D)** Durations of pauses: both, male and female yoked flies sit longer than their corresponding master fly. This is significantly more pronounced in females. **(E)** Velocity: female master flies walk faster than female yoked flies. In males this difference is not statistically significant. Female master/yoked pairs: n = 100; males master/yoked pairs: n = 130. ***p < 0.001; **p < 0.01; n.s., p > 0.05.
(TIF)

S2 Fig Male courtship. Master (blue), yoked (red), box control (turquoise) and vial-control (petrol blue). Male flies were conditioned for 20 min in the shock box and subsequently transferred to a courtship chamber where their courtship behavior was recorded for 10 min. **(A**) Wing vibration index: No differences were observed between master, yoked and control flies. **(B)** Wing vibration latency: Again no differences. **(C)** Courtship latency: No differences. Pairs: n = 20; box control: n = 22; vial control). Statistics: One-way ANOVA with post hoc (Kruskal-Wallis).
(TIF)

S3 Fig Effect of handling in the heat box. Master (black), yoked (grey). Walking activity during 10 min test phase (no heat) subsequent to 20 min of conditioning in the heat box. **(A)** No handling (master/yoked pairs: n = 58). **(B)** After handling, no difference between the groups (master/yoked pairs: n = 63). Statistics: Mann-Whitney U-Test ***p < 0.001; **p < 0.01; n.s., p > 0.05.
(TIF)
