# Supplementary material for: Inescapable Stress Changes Walking Behavior in Flies - Learned Helplessness Revisited
Source: PLoS One. 2016 Nov 22;11(11):e0167066. doi: 10.1371/journal.pone.0167066 (PMC5119826; doi:10.1371/journal.pone.0167066)
Supplement: S1 Data — (PDF) [file pone.0167066.s001.pdf]

Data Fig 2

Activity

Master			Yoked			Control		
mean	sem	n	mean	sem	n	mean	sem	n
53,90791	1,335188	220	56,27245	1,366621	220	56,22031	1,906776	174
54,50974	1,121703	220	51,03181	0,972345	220	49,97452	1,881429	174
45,81202	1,234795	220	38,23272	1,220377	220	42,34387	2,04005	174
38,13364	1,252745	220	30,55327	1,214836	220	37,56475	2,048595	174
34,23744	1,292275	220	23,94247	1,220906	220	35,99004	2,05785	174

Activity test

Master			Yoked			Control		
mean	sem	n	mean	sem	n	mean	sem	n
36,42236	1,649671	220	28,83362	1,827809	220	36,62821	1,621887	174

nop

Master			Yoked			Control		
mean	sem	n	mean	sem	n	mean	sem	n
70,55556	2,449771	220	58,05641	2,849018	220	66,39869	2,482427	174

Dur of p

Master			Yoked			Control		
mean	sem	n	mean	sem	n	mean	sem	n
5,406602	0,1766227	220	6,970671	0,2554718	220	5,726479	0,2228678	174

velocity

master			Yoked			Control		
mean	sem	n	mean	sem	n	mean	sem	n
4,217728	0,119498	220	3,741386	0,1361486	220	4,228331	0,1811059	174

Fig 3

Short pauses

m	y	C
7	5	0
7	3	4
3	3	6
2	6	2
9	7	1
6	6	4
3	10	1
8	0	1
1	5	2
1	13	11

Escape latency

Master N=220			Yoked N=220		
mean	sem	n	mean	sem	n
1,782629	0,2062057	213	2,835681	0,4413243	213
1,511737	0,1168062	213	2,144601	0,250933	213
2,051643	0,2432556	213	3,156807	0,5061138	213
1,516981	0,130489	212	3,066981	0,4296258	212
2,357143	0,3015175	210	3,040952	0,3266683	210
2,260766	0,2196969	209	2,998565	0,3251044	209
2,573558	0,3236089	208	3,244712	0,3705535	208
2,050485	0,2292041	206	3,112136	0,4041728	206
1,864878	0,2620891	205	3,071707	0,3559471	205

8	6	2	2,045813	0,2224281	203	4,14335	0,5237775	203
7	5	1	2,453769	0,3048108	199	3,601005	0,4244265	199
12	12	2	1,948731	0,1872768	197	3,388325	0,3753409	197
3	3	8	2,752551	0,3158789	196	4,144898	0,5427014	196
3	6	9	2,879793	0,3922684	193	3,761658	0,4546217	193
2	8	4	2,24579	0,2145814	190	3,224211	0,4214093	190
12	2	3	2,61164	0,3300618	189	3,511111	0,4145025	189
12	5	7	2,447872	0,2675341	188	3,575	0,4009789	188
11	5	3	2,235294	0,2084442	187	3,423529	0,3545538	187
5	1	4	2,673656	0,3129439	186	3,226882	0,282658	186
4	4	3	2,42	0,241397	185	3,848649	0,5058143	185
9	10	13	3,119672	0,3463209	183	4,245902	0,4105406	183
18	6	12	2,812778	0,2658866	180	3,224444	0,2986912	180
14	5	2	2,858427	0,3311526	178	3,781461	0,4552035	178
7	8	2	2,110286	0,159354	175	3,712	0,3949479	175
4	6	4	2,622857	0,2153284	175	3,454286	0,290165	175
15	0	0	2,578613	0,2152984	173	4,023121	0,3532179	173
0	7	4	2,375882	0,228001	170	4,095294	0,4267742	170
9	3	0	2,917365	0,2559178	167	4,352695	0,5321314	167
17	8	8	2,808696	0,2538563	161	3,870807	0,3671954	161
7	9	4	2,923899	0,3769583	159	4,922013	0,536498	159
13	1	10						
14	7	6						
5	7	4						
10	5	4						
7	6	13						
0	5	10						
0	1	7						
6	6	9						
8	6	3						
7	6	16						
2	7	3						
9	7	5						
7	6	11						
6	2	5						
9	10	6						
12	6	7						
4	2	2						
6	6	2						
8	9	5						
9	7	10						
17	3	6						
0	11	4						

17	4	9
2	7	5
4	5	0
11	5	6
2	3	10
11	15	3
18	11	5
8	7	9
4	4	6
11	8	5
8	9	5
3	12	8
7	7	7
11	5	8
14	10	7
8	10	0
7	5	8
7	4	9
5	4	0
9	6	6
4	4	2
7	10	10
0	2	1
14	9	2
3	5	0
14	5	4
2	9	6
10	3	4
9	7	3
6	1	3
4	3	3
3	0	1
17	5	3
4	5	11
10	8	5
6	2	7
7	0	3
9	6	8
5	5	2
4	11	4
6	3	4
9	6	2
12	8	3

9	10	3
1	4	3
13	7	7
8	7	4
6	8	6
7	1	2
2	10	4
4	11	2
9	7	5
3	2	10
13	9	4
4	5	5
5	3	2
6	7	2
7	4	1
12	6	9
1	6	6
10	8	4
12	9	11
12	2	4
5	7	5
11	2	7
5	6	10
5	5	5
3	8	10
4	5	12
5	0	6
4	8	16
11	7	11
5	12	3
16	6	3
3	6	10
9	2	5
8	5	4
0	6	9
10	4	7
5	11	6
19	2	7
5	6	8
3	2	4
5	9	1
2	1	1
10	1	1

11	8	6
9	7	1
4	3	9
10	1	13
11	1	3
10	5	2
12	4	2
3	8	10
10	8	2
11	5	5
2	6	3
4	8	3
1	11	2
8	3	2
5	2	3
6	2	0
5	0	7
7	9	10
8	13	2
6	4	2
3	4	9
6	5	3
6	11	3
10	5	8
4	8	6
6	3	5
7	4	5
8	14	3
12	10	3
7	7	4
8	7	7
9	7	7
6	5	5
1	1	3
13	1	1
6	1	2

Fig 4

Activity

Master			yoked/Yoked2			Control			Yoked	
mean	sem	n	mean	sem	n	mean	sem	n	mean	n
52,15677	2,170877	102	50,14624	1,582516	205	52,22348	2,593109	88		
55,03235	1,705807	102	49,92614	1,108552	205	42,62311	2,239584	88		

46,67789	1,987213	102	38,3469	1,426933	205	37,81856	2,268195	88		
37,7528	2,103402	102	26,39493	1,454633	205	36,57765	2,283151	88		
31,42046	2,199414	102	19,97353	1,394801	205	32,55379	2,346388	88		
28,80272	2,123202	102	19,5976	2,12802	103	47,10281	2,420311	88	18,9432	102
25,67585	2,085532	102	16,5433	2,070625	103	44,06827	2,291061	88	19,2442	102
23,67109	2,121267	102	16,32474	2,124656	103	36,85462	2,324608	88	19,5738	102
23,52789	2,058774	102	15,98007	2,121288	103	31,69237	2,468904	88	21,5867	102

Nop

**Master**			**yoked/Yoked2**			**Control**			**Yoked**	
mean	sem	n	mean	sem	n	mean	sem	n	mean	n
41,27723	1,265865	102	39,31373	0,9404092	205	39,14773	1,28713	88		
45,34654	1,280749	102	43,15196	0,8150575	205	35,92046	1,521643	88		
42,64357	1,4182	102	39,54412	1,111336	205	33,55682	1,662346	88		
37,91089	1,762048	102	32,82843	1,428036	205	32,71591	1,77934	88		
33,80198	1,81866	102	26,51961	1,402637	205	31,46591	1,951964	88		
33,65306	2,046528	102	25,47917	2,206317	103	39,92771	2,051152	88	26,8163	102
32,68367	2,061507	102	21,32292	2,164637	103	41,89157	1,884455	88	25,4388	102
29,62245	2,155768	102	21,14583	2,19082	103	40,24096	2,400924	88	28,2449	102
31,11225	2,272048	102	21,20833	2,233053	103	36,06024	2,329907	88	27,9184	102

Dur of p

**Master**			**yoked/Yoked2**			**Control**			**yoked**	
mean	sem	n	mean	sem	n	mean	sem	n	mean	n
3,394965	0,166019	854	3,804233	0,2059058	1604	3,661248	0,2278259	689		
2,97572	0,1192091	4580	3,482189	0,1192852	8803	4,792281	0,2374267	3161		
3,75166	0,1951608	4307	4,691142	0,239422	8067	5,559668	0,3080752	2953		
4,926038	0,2782979	3829	6,781084	0,4109766	6697	5,815735	0,3497469	2879		
6,086848	0,3600376	3414	9,092032	0,5967021	5410	6,430733	0,4215581	2769		
6,346877	0,4072749	3298	9,539431	0,692498	2422	3,974472	0,3085387	3314	9,068	2628
6,822135	0,432107	3203	11,48181	0,8533244	2089	4,005724	0,2277375	3477	9,52391	2493
7,730486	0,5299006	2903	11,58531	0,8996958	2076	4,707814	0,2927681	3340	8,54335	2768
7,374418	0,4938807	3049	11,56595	0,9096084	2088	5,682793	0,3930147	2993	8,42664	2736

Velocity

Master			yoked/Yoked2			Control			yoked	
mean	sem	n	mean	sem	n	mean	sem	n	mean	n
5,470595	0,1817987	102	5,342806	0,1427827	205	5,468805	0,2901824	88		
5,529941	0,1338472	102	5,228764	0,1035373	205	5,237792	0,2321958	88		
4,98664	0,1228208	102	4,418008	0,1381331	205	4,869245	0,2317712	88		
4,42005	0,1534628	102	3,421548	0,1519243	205	4,758825	0,2235436	88		
4,037716	0,1588522	102	3,21494	0,1700569	205	4,688178	0,1849136	88		
3,971785	0,1528657	102	3,187903	0,1913959	103	5,053508	0,1485119	88	3,3517	102
3,78731	0,1576568	102	2,605919	0,1917677	103	4,62909	0,1672062	88	3,26409	102

3,800128	0,1569181	102	2,258343	0,2134052	103	4,5147	0,2225112	88	3,22835	102
3,65188	0,1524985	102	2,178909	0,2099139	103	4,047025	0,2530244	88	3,39665	102

Fig 5

	Master			Yoked		
	PI	sem	n	PI	sem	n
pre	0,04390805	0,07366041	58	-0,09806061	0,07580957	58
tr1	0,4198276	0,06133013	58	0,1201212	0,0772881	58
te1	0,3278736	0,06156114	58	0,04333333	0,08497471	58
tr2	0,3809196	0,07085756	58	0,2538788	0,09161022	58
te2	0,2133333	0,07814304	58	0,3032727	0,08896068	58
tr2	0,4240805	0,06929662	58	0,314	0,08662154	58
postt	0,3301149	0,07766743	58	0,2377576	0,09203593	58
postt	0,1786207	0,08766676	58	0,1386667	0,09617159	58

Fig 6

master shock box no handling			yoked shock box no handling		
mean	sem	n	mean	sem	n
33,72393	3,260893	38	20,00171	3,179766	38
**master with handling**			**yoked with handling**		
36,24853	3,167132	34	24,52304	3,396003	34
**master with transfer**			**yoked with transfer**		
53,63636	3,554584	29	56,02955	2,89311	29

Fig 7

Activity

Master			Yoked			Box-Control		
mean	sem	n	mean	sem	n	mean	sem	n
60,82895	3,886462	20	59,51754	4,009265	20	60,38636	6,989436	22
57,93947	3,171741	20	50,51316	3,376628	20	53,35	6,175293	22
42,36316	3,980537	20	31,33333	3,767304	20	43,66818	6,561628	22
37,73772	3,901376	20	24,77719	3,785399	20	37,14394	6,73276	22
34,72807	4,119574	20	20,74474	3,525763	20	32,88788	6,71816	22

Chamber exit latency

Master	Yoked	Control
4	3	8
11	10	12
20	29	4
6	3	6

Courtship index

Master			Yoked		
Index	sem	n	Index	sem	n
0,90278	0,04857915	20	0,883275	0,04310166	20
**Box-Control**			**Vial-Control**		

7	120	3	Index	sem	n	Index	sem	n
3	180	180	0,800895	0,06343138	27	0,783395	0,07755023	27
12	3	180						
3	16	8						
3	3	5						
30	180	20						
53	5	9						
180	9	5						
4	180	8						
2	3	60						
26	180	120						
10	180	180						
6	180	15						
13	24	45						
11	180	8						
7	180	6						
5	9	3						
3	16	10						
4	24	4						
8	16	2						
15	24	12						
180	12	5						
8	5	2						
3	2	2						
5	15	8						
12	180	2						
3	3	20						
8	7	180						
8	12	1						
180	11	5						
5	29	4						
8	4	4						
180	10	34						
22	40	2						
	180	6						
	94	12						
	9	6						
	6							
	180							
	43							

Fig 8

Activity [%]

Master	yoked	box control	vial control
79,66945	72,6778	87,86978	87,11519
75,7596	60,36728	78,01336	89,71285
35,25542	60,88481	82,39399	87,18197
76,77129	66,92154	93,8798	33,51586
35,38564	88,68447	95,05175	94,3005
76,71118	41,01168	81,19533	92,85809
73,23539	15,70284	80,40067	91,13857
69,47579	66,0768	88,35726	91,16528
6,317195	9,949917	85,35226	97,59265
74,02671	25,97663	57,66945	94,4975
74,50752	39,36227	91,9399	93,55927
52,57429	71,17864	90,01669	76,22371
45,58598	60,51085	76,38397	73,28548
54,90484	53,18531	41,46912	74,11353
29,42571	64,197	87,10851	86,14357
55,78631	38,5409	93,20868	48,16027
50,14023	83,94324	81,42571	94,82471
55,51252	59,72621	79,40568	93,41903
36,55426	48,88815	62,95493	87,24207
25,96661	67,99666	87,68948	92,69783

Time in rim zone [%]

Master	Yoked	box control	vial control
68,12688	63,38898	62,63773	73,77629
36,35392	55,86644	57,04841	58,47413
32,55759	54,1202	58,57763	65,48915
50,4975	54,11352	70,38397	22,89149
30,83472	87,47246	63,33556	76,995
75,33556	20,37062	80,19366	83,05175
69,10184	14,29048	74,64107	69,32221
66,16361	59,71619	85,70952	78,77129
5,232053	6,520868	71,75292	83,02504
63,5192	13,68281	54,45075	71,95326
70,64774	36,99833	66,17028	72,94491
43,79299	69,98331	86,78798	61,74291
38,55426	51,6394	71,76294	65,61603
37,98665	40,12688	34,08681	67,85309
27,46578	50,34057	83,46912	79,9399
49,06511	32,09683	86,10684	40,38063
33,02838	61,90985	74,32053	44,61102
43,65276	47,39232	61,69616	85,49249
32,42404	41,10851	40,35392	74,86811
12,14023	46,9182	76,80801	84,06344

Fig 9

Activity

Single reared			Group reared		
mean	sem	n	mean	sem	n
51,95852	1,855613	113	42,18309	1,556478	174

Nop

Single reared			Group reared		
mean	sem	n	mean	sem	n
8,057576	0,2662352	113	7,576355	0,2205686	174

Dur of p

Single reared			Group reared		
mean	sem	n	mean	sem	n
3,562686	0,1898869	27684	4,643629	0,1974084	19153

Velocity

Single reared	Group reared

mean	sem	n	mean	sem	n
4,801104	0,2014233	113	4,512307	0,1728058	174

Fig 10

Activity

Group reared Master Training			Group reared Yoked			Single reared Master			Single reared Yoked		
mean	sem	n	mean	sem	n	mean	sem	n	mean	sem	n
54,50376	1,78663	118	56,97396	1,87225	118	57,31818	1,959482	103	55,70068	1,963353	103
56,53359	1,290564	118	51,17448	1,396828	118	54,2064	1,680871	103	46,69184	1,719465	103
39,41629	1,489204	118	37,90313	1,590351	118	46,41549	1,927948	103	36,07959	1,99992	103
35,69474	1,573448	118	30,95729	1,590828	118	41,20673	1,872261	103	30,70102	1,789289	103
35,69474	1,599244	118	24,60833	1,598064	118	39,21212	1,911975	103	29,55442	1,874509	103

Activity Test

Master Group reared			Yoked group reared			Master single reared			Yoked single reared	
mean	sem	n	mean	sem	n	mean	sem	n	mean	n
29,63291	1,912444	118	23,1315	1,850689	118	37,62647	2,077387	103	31,8814	103

dur of p

Group reared Master			Group reared Yoked			Single reared Master			Single reared Yoked	
mean	sem	n	mean	sem	n	mean	sem	n	mean	n
6,857523	0,295144	7265	9,032847	0,4047058	6025	5,162645	0,2055727	7394	5,8535	7122

nop

Group reared Master			Group reared Yoked			Single reared Master			Single reared Yoked	
mean	sem	n	mean	sem	n	mean	sem	n	mean	n
61,5678	3,097605	118	51,05932	3,017028	118	72,4902	2,920902	103	69,8235	103

Velocity

Group reared Master			Group reared Yoked			Single reared Master			Single reared Yoked	
mean	sem	n	mean	sem	n	mean	sem	n	mean	n
3,813834	0,1479215	118	3,290366	0,1558034	118	3,760949	0,1048936	103	3,54758	103
