# Supplementary figures and images for: Inescapable Stress Changes Walking Behavior in Flies - Learned Helplessness Revisited
Source: PLoS One. 2016 Nov 22;11(11):e0167066. doi: 10.1371/journal.pone.0167066 (PMC5119826; doi:10.1371/journal.pone.0167066)

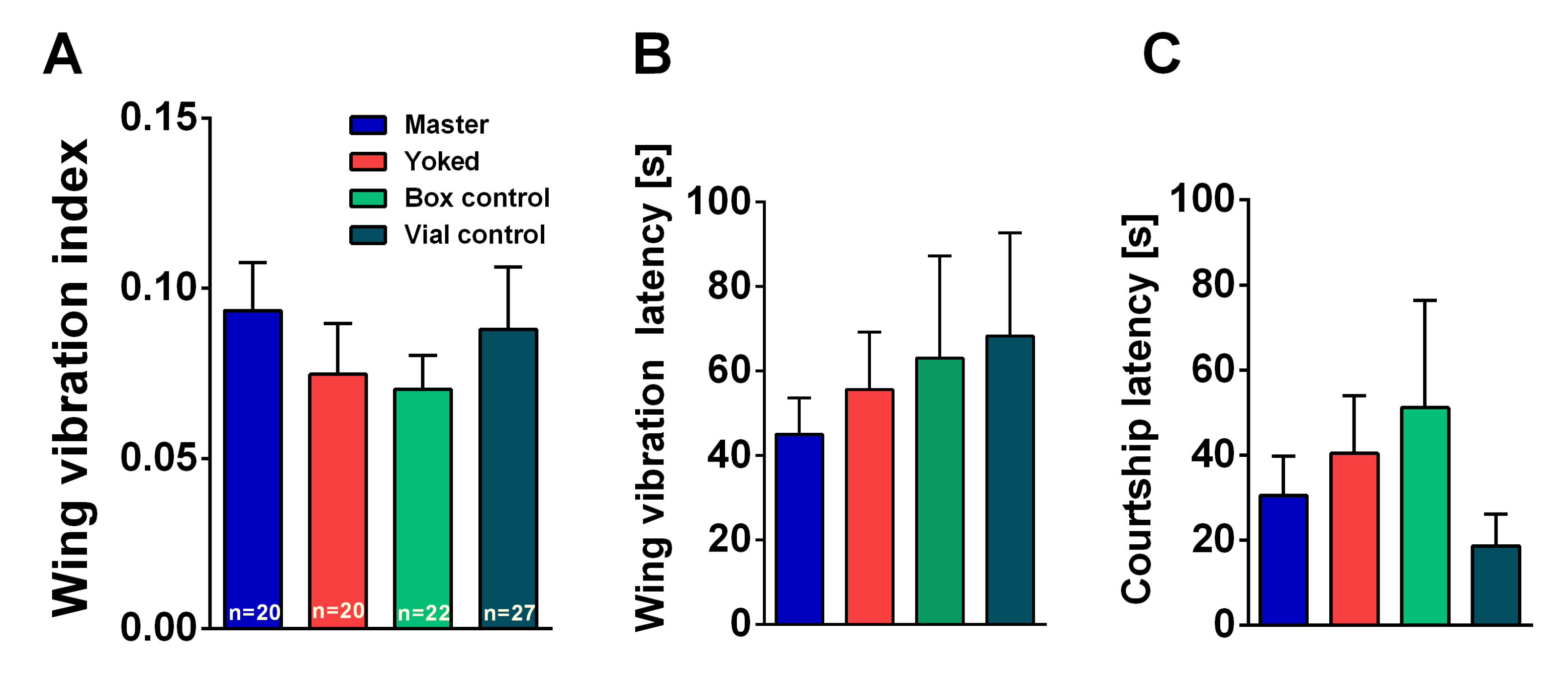

Supplement: S2 Fig — Master (blue), yoked (red), box control (turquoise) and vial-control (petrol blue). Male flies were conditioned for 20 min in the shock box and subsequently transferred to a courtship chamber where their courtship behavior was recorded for 10 min. (A) Wing vibration index: No differences were observed between master, yoked and control flies. (B) Wing vibration latency: Again no differences. (C) Courtship latency: No differences. Pairs: n = 20; box control: n = 22; vial control). Statistics: One-way ANOVA with post hoc (Kruskal-Wallis). (TIF) [file pone.0167066.s003.tif]

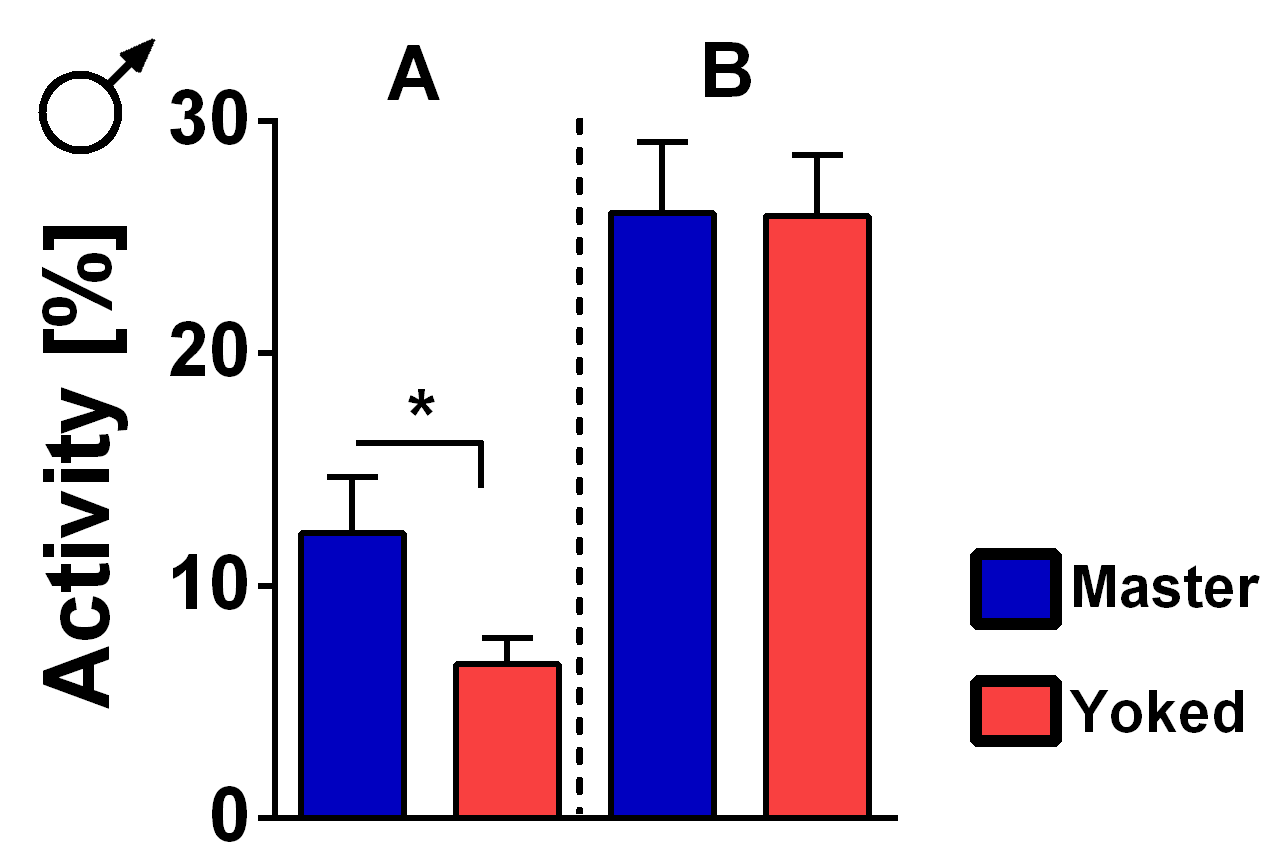

Supplement: S3 Fig — Master (black), yoked (grey). Walking activity during 10 min test phase (no heat) subsequent to 20 min of conditioning in the heat box. (A) No handling (master/yoked pairs: n = 58). (B) After handling, no difference between the groups (master/yoked pairs: n = 63). Statistics: Mann-Whitney U-Test ***p < 0.001; **p < 0.01; n.s., p > 0.05. (TIF) [file pone.0167066.s004.tif]
